# Evidence for the contribution of *COMT* gene Val158/108Met polymorphism (rs4680) to working memory training‐related prefrontal plasticity

**DOI:** 10.1002/brb3.1523

**Published:** 2020-01-09

**Authors:** Wan Zhao, Ling Huang, Yang Li, Qiumei Zhang, Xiongying Chen, Wenjin Fu, Boqi Du, Xiaoxiang Deng, Feng Ji, Yu‐Tao Xiang, Chuanyue Wang, Xiaohong Li, Qi Dong, Chuansheng Chen, Susanne M. Jaeggi, Jun Li

**Affiliations:** ^1^ State Key Laboratory of Cognitive Neuroscience and Learning & IDG/McGovern Institute for Brain Research Beijing Normal University Beijing China; ^2^ School of Mental Health Jining Medical University Jining China; ^3^ The National Clinical Research Center for Mental Disorders & Beijing Key Laboratory of Mental Disorders & Beijing Institute for Brain Disorders Center of Schizophrenia Beijing Anding Hospital Capital Medical University Beijing China; ^4^ Faculty of Health Sciences University of Macau Taipa China; ^5^ Department of Psychological Science University of California Irvine CA USA; ^6^ School of Education & Department of Cognitive Sciences University of California Irvine CA USA

**Keywords:** *COMT*, fMRI, gene polymorphism, randomized controlled trial, working memory training

## Abstract

**Background:**

Genetic factors have been suggested to affect the efficacy of working memory training. However, few studies have attempted to identify the relevant genes.

**Methods:**

In this study, we first performed a randomized controlled trial (RCT) to identify brain regions that were specifically affected by working memory training. Sixty undergraduate students were randomly assigned to either the adaptive training group (*N* = 30) or the active control group (*N* = 30). Both groups were trained for 20 sessions during 4 weeks and received fMRI scans before and after the training. Afterward, we combined the data from the 30 participants in the RCT study who received adaptive training with data from 71 additional participants who also received the same adaptive training but were not part of the RCT study (total *N* = 101) to test the contribution of the *COMT* Val158/108Met polymorphism to the interindividual difference in the training effect within the identified brain regions.

**Results:**

In the RCT study, we found that the adaptive training significantly decreased brain activation in the left prefrontal cortex (TFCE‐FWE corrected *p* = .030). In the genetic study, we found that compared with the Val allele homozygotes, the Met allele carriers' brain activation decreased more after the training at the left prefrontal cortex (TFCE‐FWE corrected *p* = .025).

**Conclusions:**

This study provided evidence for the neural effect of a visual–spatial span training and suggested that genetic factors such as the *COMT* Val158/108Met polymorphism may have to be considered in future studies of such training.

## Graphical abstract

The adaptive spatial span training decreased brain activation in the prefrontal and parietal cortex. The *COMT* Val158Met polymorphism modulated the left frontal plasticity induced by training.

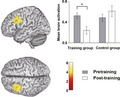

## 1 INTRODUCTION

Working memory, an ability to maintain and manipulate information for a short period of time (Miyake & Shah, 1999), has been reported to be trainable (Danielsson, Zottarel, Palmqvist, & Lanfranchi, 2015). However, only a limited number of studies have explored the neural mechanism for working memory training effect (Beatty et al., 2015; Dahlin, Neely, Larsson, Bäckman, & Nyberg, 2008; Schweizer, Grahn, Hampshire, Mobbs, & Dalgleish, 2013). As far as memory span training is concerned, there are fewer studies and the results are contradictory. To the best of our knowledge, there has been only one fMRI randomized controlled trial (RCT; Brehmer et al., 2011) that randomly assigned 23 older adults aged from 60 to 70 years to either the experimental group that received adaptive training on seven memory span tasks (*N* = 12) or to the control group that received low‐level practice on the same set of span tasks (*N* = 11). The authors reported that the training led to decreased brain activation within the frontal cortex. By contrast, two earlier fMRI studies of working memory training, both of which had smaller sample sizes and lacked a control group (Olesen, Westerberg, & Klingberg, 2004; Wexler, Anderson, Fulbright, & Gore, 2000), found increased brain activity in the frontal cortex and parietal cortex after the training.

One possible reason for these inconsistent results is the sizable interindividual difference in training effects as pointed out by previous researchers (Burki, Ludwig, Chicherio, & Ribaupierre, 2014; Schubert, Strobach, & Karbach, 2014). Recently, researchers have speculated on the genetic factors involved in such interindividual differences (Bellander et al., 2011; Brehmer et al., 2009; Panizzutti, Hamilton, & Vinogradov, 2013; Soderqvist, Matsson, Peyrard‐Janvid, Kere, & Klingberg, 2014). Dopamine‐related genes may be promising candidates due to the fundamental importance of dopamine to working memory (Cools & D'Esposito, 2011). In fact, PET studies have shown that working memory training could alter brain dopamine activity (Backman et al., 2011; McNab et al., 2009). The Val158/108Met polymorphism (i.e., rs4680) in the gene coding for catechol‐O‐methyltransferase (COMT), an enzyme that breaks down the dopamine in the synapse, plays a pivotal role in regulating dopamine level in the prefrontal cortex (Tunbridge, Harrison, & Weinberger, 2006). Individuals with the Met allele have relatively lower *COMT* activity and accordingly higher dopamine levels in the synapse than individuals with the Val allele (Chen et al., 2004; Lotta et al., 1995). Many studies have confirmed the important roles of this polymorphism in working memory and its underlying brain basis (Aguilera et al., 2008; Bellander et al., 2015; Bruder et al., 2005; Farrell, Tunbridge, Braeutigam, & Harrison, 2012; Jin et al., 2016; Kennedy et al., 2011; Wang et al., 2013). Within the field of working memory training, a previous behavior study of a Caucasian sample (aged 20–80) associated the Met allele with smaller behavioral performance gains in working memory (Bellander et al., 2015). However, it is still unknown whether the same polymorphism is linked to brain plasticity resulting from working memory training.

Given the limited and conflicting results regarding the neural effects of memory span training, the current study first aimed to identify the brain regions that were specifically affected by memory span training using the fMRI technique. We recruited 60 undergraduate students who were randomly assigned to either the adaptive training group (*N* = 30, receiving adaptive training on a visual–spatial span task) or the active control group (*N* = 30, receiving practice on an easy version of the same visual–spatial span task). Both groups were trained for 20 sessions over the course of 4 weeks. We hypothesized that training would decrease brain activation, specifically in the frontal and parietal cortices because of the importance of these regions for working memory (Brehmer et al., 2011; Olesen et al., 2004; Salmi, Nyberg, & Laine, 2018). Afterward, we investigated the contribution of *COMT* gene Val158/108Met polymorphism to training‐related activation changes in the identified regions. We combined the data from the 30 participants in the RCT study who received adaptive training with data from 71 additional participants who also received the same adaptive training but were not part of the RCT study. We hypothesized that *COMT* Val158/108Met polymorphism would modulate the training‐related brain plasticity, especially in the prefrontal cortex.

## 2 MATERIALS AND METHODS

The protocol of this study was reviewed and approved by the Institutional Review Board of the Institute of Cognitive Neuroscience and Learning at Beijing Normal University. This study had been registered at the Chinese Clinical Trial Registry (http://www.chictr.org.cn; chiCTR‐INR‐17011728). All subjects gave written informed consent for this study.

### 2.1 Subjects

A total of 60 healthy undergraduate students were recruited from Beijing Normal University through an Internet advertisement. Each subject received an unstructured clinical interview with an experienced psychiatrist to screen for any personal or family history of mental disorders. Subjects were randomly allocated to either the adaptive training group (*N* = 30) or the nonadaptive training control group (*N* = 30) according to a computer‐generated list of random numbers. For both groups, the training consisted of 20 sessions conducted in our neuropsychological laboratory over the course of 4 weeks (five sessions per week, no more than one session per day). All subjects received the same set of neuropsychological assessments and fMRI scans before and after training, and they received 500 Chinese Yuan (CNY) for their participation. CONSORT diagram for randomized controlled trials is shown in the Figure S1.

In addition to the above sample for the RCT, we recruited an additional sample of 71 undergraduate students from the same university. These subjects received the same training as the 30 subjects in the adaptive training group. The combined 101 subjects who received adaptive training were used to examine the effect of *COMT* gene Val158/108Met polymorphism on brain plasticity related to the training.

### 2.2 Task for training and assessment

The working memory training was developed based on a visual–spatial span task (see Figure S2A). Stimuli were green‐colored squares presented sequentially in a 5 × 5 empty grid on a computer screen. Each stimulus was presented for 500 ms, with a 500 ms interstimulus interval. After the presentation of the last stimulus, there was a 1,000 ms intermission screen followed by an empty grid. Subjects were required to recall the sequence of locations by clicking on the appropriate squares in the empty grid. Difficulty level was determined by the number of the stimuli that had to be remembered. For the adaptive training group, the training started with 3 stimuli during the first session, and the number of stimuli was automatically increased by 1 if subjects made 5 continuous correct responses at their current difficulty level. For the subsequent sessions, the training started with two stimuli fewer than the highest number of stimuli during the previous session. For the control group, the number of stimuli was kept at 3 throughout the training sessions. All subjects were required to complete 80 trials in each training session (lasting 30–40 min). Finally, we revised this training task to create an assessment version to measure training gains on memory span. Two trials were given at each span length in the assessment version. Testing ceased when the subject failed at both trials. We used the length of the longest span recalled correctly to reflect memory span. All subjects finished this task both before and after the training.

### 2.3 Task for fMRI scan

The fMRI task included the working memory condition and the baseline condition (see Figure S2B). The stimuli were presented in cue–probe pairs. The working memory condition used the same cue stimuli as those used for training (see above), except that the number of stimuli was set at 5. The probe stimulus was a green‐colored Arabic number within the empty grid. Subjects were asked to judge if the Arabic number indicated the correct order of the cue stimuli. For the baseline condition, the cue stimuli were 5 red‐colored squares presented in the same order (top left corner → top right corner → bottom right corner → bottom left corner → center) across all trials. The probe stimuli for the baseline condition were the same as those for the working memory condition except that the Arabic number was red‐colored. The fMRI task included 72 working memory trials and 36 baseline trials (108 trials in total). Each trial started with a fixation cross for 500 ms, followed by 5 sequentially presented cue stimuli. Each cue stimulus was presented for 500 ms, with a 500 ms interstimulus interval. The total time for the presentation of cue stimuli was 4,500 ms. After a 1,000 ms intermission screen, probe stimuli were presented for 2,000 ms, during which subjects made their responses using a fiber‐optic response box. Subjects pressed the left button if the Arabic number at a given location correctly reflected the sequence number of the cue stimuli and pressed the right button if the Arabic number was incorrect.

### 2.4 fMRI data acquisition

All subjects were scanned both before and after the training on a Siemens TIM Trio 3T scanner (Siemens) at the Brain Imaging Center of Beijing Normal University. During scanning, subject's head was snugly fixed with straps and foam pads to restrict head movement. Functional images during the performance of the spatial span task as mentioned above were collected axially using the following echo‐planar imaging (EPI) sequence: repetition time (TR) = 2,000 ms; echo time (TE) = 30 ms; flip angle (FA) = 90°; field of view (FOV) = 200 × 200 mm^2^; matrix size = 64 × 64; axial slices = 31; 4.0 mm slice thickness without gap (i.e., interleaved scan); voxel size = 3.1 × 3.1 × 4.0 mm^3^.

### 2.5 fMRI data preprocessing

Data preprocessing was implemented using Statistical Parametric Mapping software (SPM12, Wellcome Department of Cognitive Neurology). Preprocessing included realignment (correcting for head movement, any subject with more than 2 mm translation or 2° rotation was excluded), normalization (to the Montreal Neurological Institute [MNI] space), resampling (to a voxel size of 3 × 3 × 3 mm^3^), and spatial smoothing (with 8 mm full‐width at half maximum (FWHM) of the Gaussian smoothing kernel). We focused our analysis on the cue phase (from the start of the cue stimuli to the start of the probe stimuli) of the correct trials which covered cognitive processing of both encoding and maintenance during working memory. In the first‐level (within‐subjects) analysis, we used task condition (working memory vs. baseline) as a predictor to produce brain activation images for each subject at each time point. In this analysis, a high‐pass filter at 128 s was used to remove noise associated with low‐frequency confounds. The resulting images were entered into the second‐level (between‐subjects) data analysis.

### 2.6 Genotyping

Genomic DNA for subjects who received adaptive training (*N* = 101) was extracted using the standard method. *COMT* gene Val158/108Met polymorphism was genotyped using TaqMan allele‐specific assays on the 7900HT Fast Real‐Time PCR System (Applied Biosystems). The sample success rate for this SNP was 100%. The reproducibility of the genotyping was 100% according to a duplicate analysis of 30% of the genotypes.

### 2.7 Data analysis

Analyses on both behavioral data and demographic data were performed using SPSS version 22.0. One‐way ANOVAs and chi‐square tests were used to compare the two groups in terms of demographic variables and baseline behavioral performance on all tasks. We then performed the repeated‐measures ANOVA to explore the training‐specific effects on behavioral performance. In this analysis, time (pretraining vs. post‐training) was entered as a within‐subject factor, and group (adaptive training vs. control) was entered as a between‐subject factor. Significance level was set at *p* < .05 (two‐sided). Effect sizes were calculated using Cohen's *d*. Significant time × group interaction effect was followed up by post hoc analysis using paired sample *t* test.

For the between‐subjects analysis on fMRI RCT data, we first used two‐sample *t* tests to investigate whether pretraining brain activation was comparable between the two groups. Next, we used mixed factorial ANOVA on the contrast images (working memory vs. baseline) that were produced at the end of the fMRI data preprocessing to identify brain regions showing training‐specific activation changes. In this analysis, time (pretraining vs. post‐training) was entered as a within‐subject factor and group (adaptive training vs. control) was entered as a between‐subject factor. Based on previous findings (Dahlin, Neely, Larsson, Bäckman, et al., 2008; Kelly & Garavan, 2005; Opitz, Schneiders, Krick, & Mecklinger, 2014), we limited this analysis to task‐relevant brain regions. Task‐relevant brain regions were produced using one‐sample *t* test on the contrast images (working memory vs. baseline) of all subjects at pretraining. A Montreal Neurological Institute (MNI) space gray matter mask (GRETNA software, https://www.nitrc.org/projects/gretna/) was applied to isolate the task‐relevant brain regions in gray matter. For the regions showing significant time × group interaction effect, we conducted post hoc analysis on the extracted mean brain activation value using paired sample *t* test.

Finally, we also used mixed factorial ANOVA to test the contribution of *COMT* Val158/108Met to the training‐related brain activation changes within the significant cluster at the left prefrontal cortex that was identified in the above analysis. In this analysis, time (pretraining vs. post‐training) was entered as a within‐subjects factor and genotype (Met carriers vs. Val/Val) was entered as a between‐subjects factor.

Within the above fMRI data analyses, the threshold‐free cluster enhancement (TFCE) approach (10,000 random permutations) (Smith & Nichols, 2009) with thresholding set at combined peak‐cluster‐level *p* < .05 family‐wise error (FWE) was performed using the TFCE toolbox for SPM (http://dbm.neuro.uni-jena.de/tfce) to correct for multiple comparisons.

## 3 RESULTS

**Figure 1:**
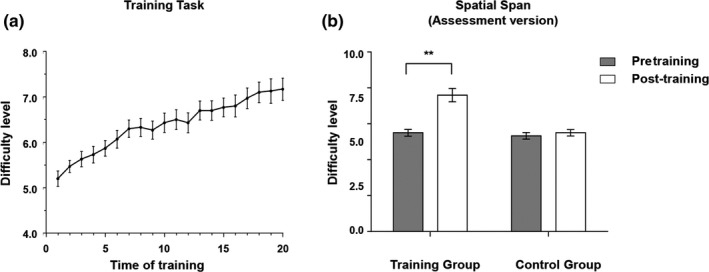
Behavioral performance (means and standard deviations) of the adaptive training group on the spatial span task. For the training version of the task, the training group showed gradual improvement during the 20 training sessions (Panel a). For the assessment version of the trained spatial span task, the training group showed greater improvement compared with the control group (Panel b)

The two groups did not differ in demographic variables and pretraining performance on the memory span task (*P*s > 0.05, Table 1). During the training, the adaptive training group showed gradual improvement in their performance on the memory span task (see Figure 1a). The repeated‐measures ANOVA analysis found a significant time × group interaction effect (*F* = 20.73, *p* < .001, Cohen's *d* = 1.02, Figure 1b) on the assessment version of the trained spatial span task. Post hoc analysis showed that the performance of the training group was significantly improved (*t* = −5.89, *p* < .001), whereas that of the control group showed no significant change (*t* = −0.72, *p* = .475).

**Table 1:** Demographic variables across groups in the randomized, controlled study

	Mean ± *SD*		*F* or *χ* ^2^	*p*
	Training group	Control group		
Number of subjects	30	30		
Gender (male/female)	3/27	5/25	0.58	.448
Age (years)	21.40 ± 2.28	21.5 ± 1.98	0.06	.810
Education (years)	15.03 ± 1.56	15.40 ± 1.45	0.89	.351
IQ^a^	130.77 ± 5.44	128.03 ± 5.45	3.25	.077

a Full scale IQ, as measured by Wechsler Adult Intelligence Scale.

**Figure 2:**
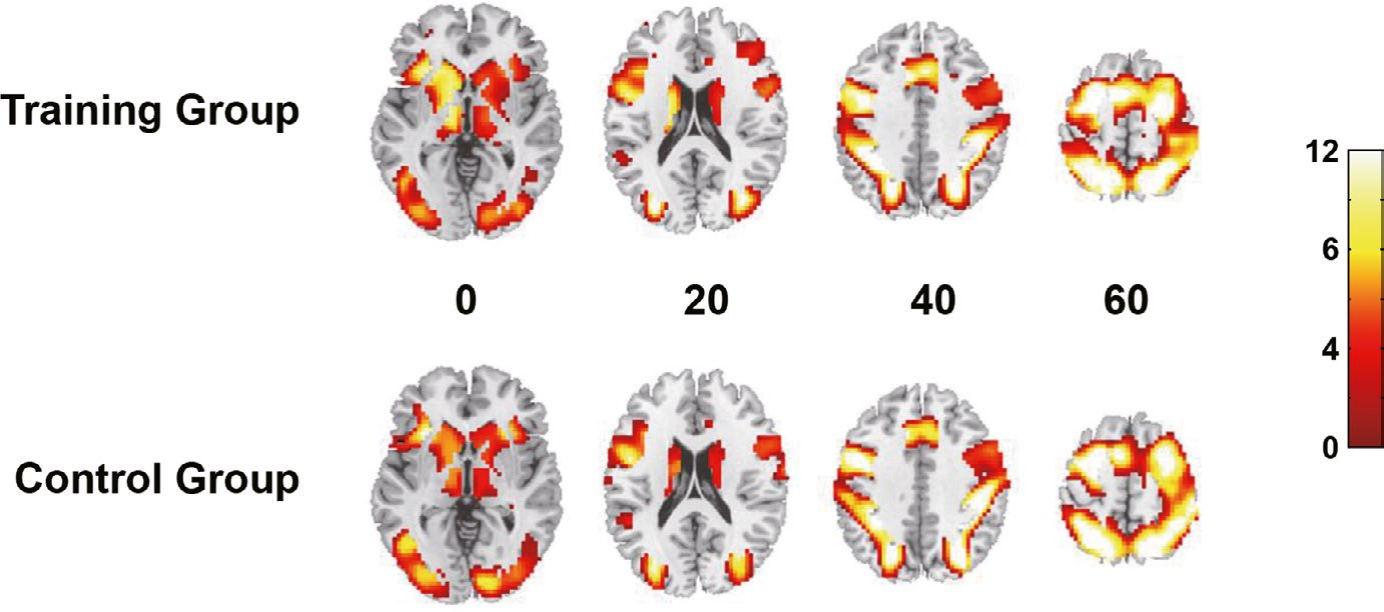
Whole‐brain activation of the two groups on the spatial span task at pretraining. There was no significant difference between the groups (TFCE‐FWE corrected *p* > .05)

Due to their excessive head motions (>2° or 2 mm) at either pre‐ or post‐training scan, five subjects in the training group and five subjects in the control group were excluded from the final fMRI analysis on the training effect. The remaining 50 subjects (25 for each group) did not show group differences in any of the demographic variables (all *p* > .05). The accuracy on the fMRI version of the span task was high and did not differ by group or time point (all *p* > .05). The two groups also did not differ in whole‐brain activation before training (FWE corrected *p* > .05, see Figure 2). These results indicated that the two groups were comparable in terms of background characteristics.

**Figure 3:**
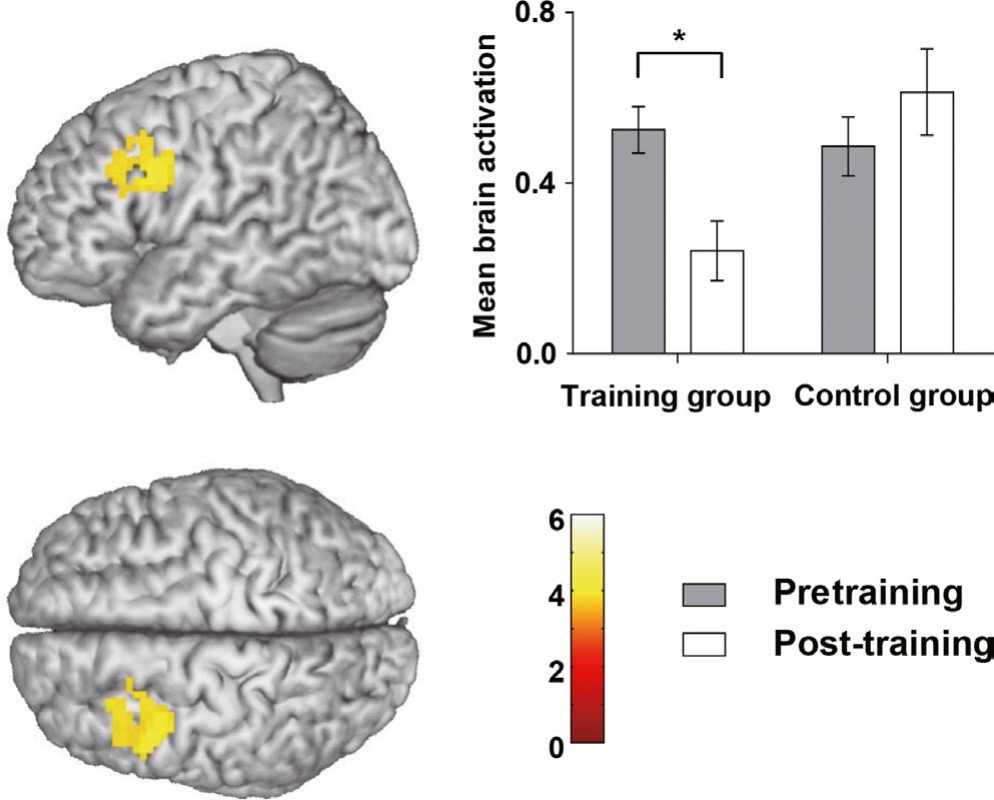
Brain activation (means and standard deviations) before and after training by group. Brain activation decreased significantly in the training group but did not change significantly in the control group in the left prefrontal cortex (TFCE‐FWE corrected *p* < .05)

The mixed factorial ANOVA showed a significant time × group interaction in the left prefrontal cortex (cluster size = 147 voxels, TFCE‐FWE corrected *p* = .030). Post hoc analysis showed that brain activation decreased significantly after training in the training group (*t* = 4.13, *p* < .001), but not in the control group (*t* = −1.45, *p* = .160; see Figure 3).

**Figure 4:**
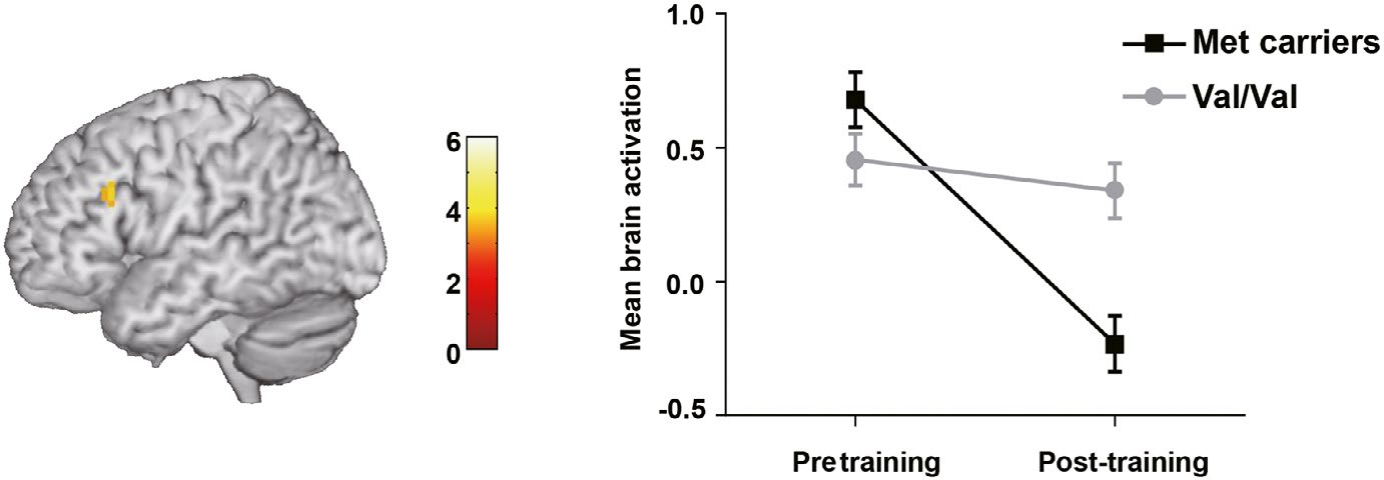
Brain activation (means and standard deviations) of the left prefrontal cortex before and after training by *COMT* Val158/108Met polymorphism (TFCE‐FWE corrected *p* < .05)

Then, we tested the effect of *COMT* Val158/108Met polymorphism on the fMRI plastic changes within the left prefrontal cortex. No significant deviation from the Hardy–Weinberg equilibrium (*p* > .05) was found for this polymorphism. Five subjects were excluded due to their excessive head motions (>2° or 2 mm) at either pre‐ or post‐training scan. The remaining 96 subjects showed no significant differences between the two genotype groups in the demographic variables (*P*s > 0.05; see Table S1). Within the significant cluster (cluster size = 147 voxels) in the left prefrontal cortex that showed significant training‐specific brain activation change in the above analysis, we found a significant genotype × time interaction (cluster size = 7 voxels, TFCE‐FWE corrected *p* = .025; see Figure 4). Post hoc analysis showed that the Met allele carriers' prefrontal activation decreased significantly after the training (*t* = 7.36, *p* < .001), whereas subjects with the Val/Val genotype did not change much (*t* = 1.99, *p* = .053).

## 4 DISCUSSION

In the current study, we first conducted a randomized, controlled fMRI study to examine the effect of memory span training on brain plasticity, and we then examined the contribution of *COMT* Val158/108Met polymorphism to the interindividual difference in training‐related plasticity. We found that the training decreased brain activation specifically within the left prefrontal cortex and that *COMT* Val158/108Met polymorphism could modulate this plasticity within the left prefrontal cortex.

Our finding that adaptive spatial span training significantly decreased brain activation within the left prefrontal cortex is consistent with that of Brehmer et al.'s (2011) RCT study that used a similar memory span intervention. Other types of working memory training such as updating training using a dual n‐back task have also consistently reported training‐induced decreases in brain activation within the frontal cortex (Dahlin, Neely, Larsson, Backman, & Nyberg, 2008; Salminen, Kuhn, Frensch, & Schubert, 2016; Schneiders, Opitz, Krick, & Mecklinger, 2011), the brain region crucial for working memory (Baier et al., 2010; Ekman, Fiebach, Melzer, Tittgemeyer, & Derrfuss, 2016). Indeed, a recent meta‐analysis (Salmi et al., 2018) summarizing the neural effects of working memory training provides further support to the conclusion that working memory training increases neural efficiency (i.e., activation decrease) in the frontal cortex. However, early studies with very small sample sizes reported that intensive training with memory span tasks increased brain activation in the frontal cortex (Olesen et al., 2004; Wexler et al., 2000). One possible explanation for this discrepancy is that without an RCT design that also includes an active control group, there could be some nonspecific effects on brain activation, such as test–retest effects, expectancy, and/or repeated practice effects. For example, previous studies have also reported that low‐level repeated practice on span tasks was associated with increased brain activation within the frontal lobes (Jolles, Buchem, Rombouts, & Crone, 2012; Jolles, Grol, Van Buchem, Rombouts, & Crone, 2010).

Another important result of this study was that the Met allele of the *COMT* Val158/108Met polymorphism was associated with better prefrontal plasticity (as indicated by a greater decrease in training‐related prefrontal activation). This is in contrast with Bellander et al.'s (2015) behavioral study of Caucasian young and old adults (aged 20–80 years), which showed that the Met allele was associated with weaker working memory plasticity (as indicated by less training gains). At least three factors have to be considered. First, the sample in the current study was Han Chinese, whose frequency of the Met allele was much lower than that in Caucasians (0.5) (Bellander et al., 2015). The association of the *COMT* Val158/108Met polymorphism has been found to vary by population. Although most studies in the Caucasian adults linked the Met allele with better working memory performance (Aguilera et al., 2008; Bellander et al., 2015; Bruder et al., 2005; Farrell et al., 2012; Kennedy et al., 2011), previous studies in healthy Han Chinese samples have reported the association in the opposite direction (Jin et al., 2016; Wang et al., 2013). Second, findings on the association between the *COMT* Val158/108Met polymorphism and prefrontal activation during working memory are not as consistent as findings on the association between the same polymorphism and working memory performance. A recent meta‐analysis on 14 independent studies that involved a total of 920 healthy subjects found no significant association between the *COMT* Val158/108Met polymorphism and prefrontal activation (Nickl‐Jockschat, Janouschek, Eickhoff, & Eickhoff, 2015), which is consistent with our current finding on the relationship between this polymorphism and the prefrontal function before the training. Finally, the sample in the current study was much younger than the sample in Bellander et al.'s study (2015). The role of the *COMT* Val158/108Met polymorphism in brain function varies by age due to the nonlinear effect of age on working memory (Sander, Lindenberger, & Werkle‐Bergner, 2012), the age‐dependent relationship between the *COMT* activity and the prefrontal dopamine level (Tunbridge et al., 2006, 2007), and the inverted U‐shaped relationship between prefrontal dopamine level and cognitive function (Cools & D'Esposito, 2011). Specifically, the developmental trajectories of both working memory and *COMT* activity in the prefrontal cortex are of the same pattern—increasing during childhood and adolescence, peaking at young adulthood, and declining gradually after that (Sander et al., 2012; Tunbridge et al., 2006). Because our current study had a much younger sample (whose working memory was still developing and approaching its peak) than did Bellander et al.'s study (2015; whose subjects' working memory had peaked and was declining), we observed different effects of the *COMT* Val158/108Met polymorphism on prefrontal plasticity. It needs to be mentioned that two independent studies (Dumontheil et al., 2011; Wahlstrom, Collins, White, & Luciana, 2010) in Caucasian adolescence and young adults (aged 6–20 and 9–25, respectively) have consistently reported steeper working memory developmental plasticity of the Met allele, which is similar to our current finding that the Met allele was associated with better prefrontal plasticity induced by training.

In conclusion, this study provided evidence for the neural effect of a visual–spatial span training and identified a genetic factor for interindividual differences in the training‐related neural effect. We found that the *COMT* Val158/108Met polymorphism modulated the prefrontal cortex plasticity induced by working memory training. These results suggest possible gene‐based personalized cognitive training in the future.

## CONFLICT OF INTEREST

The authors declare that they have no conflict of interest.

## Data Availability

The data that support the findings of this study are openly available at the Chinese Clinical Trial Registry (http://www.chictr.org.cn; chiCTR‐INR‐17011728).
