# Supplementary figures and images for: Evidence for the contribution of COMT gene Val158/108Met polymorphism (rs4680) to working memory training‐related prefrontal plasticity
Source: Brain Behav. 2020 Jan 9;10(2):e01523. doi: 10.1002/brb3.1523 (PMC7010579; doi:10.1002/brb3.1523)

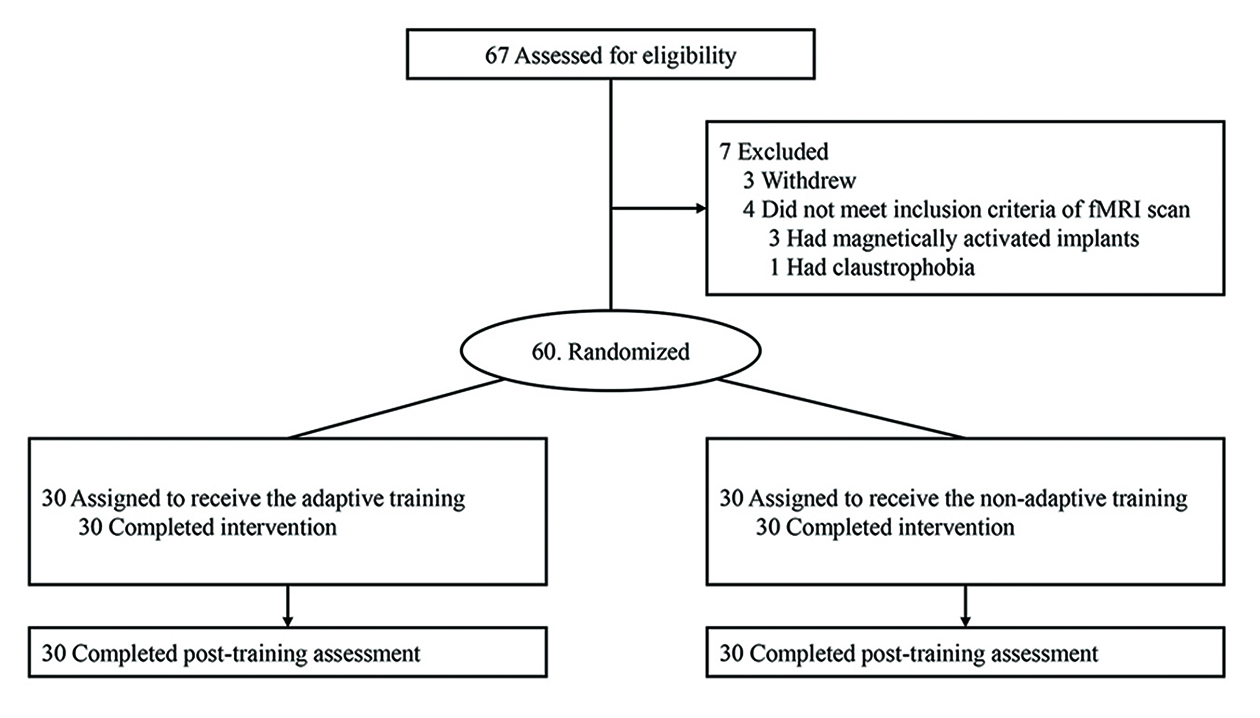

Supplement: Supplementary file 1 [file BRB3-10-e01523-s001.tif]

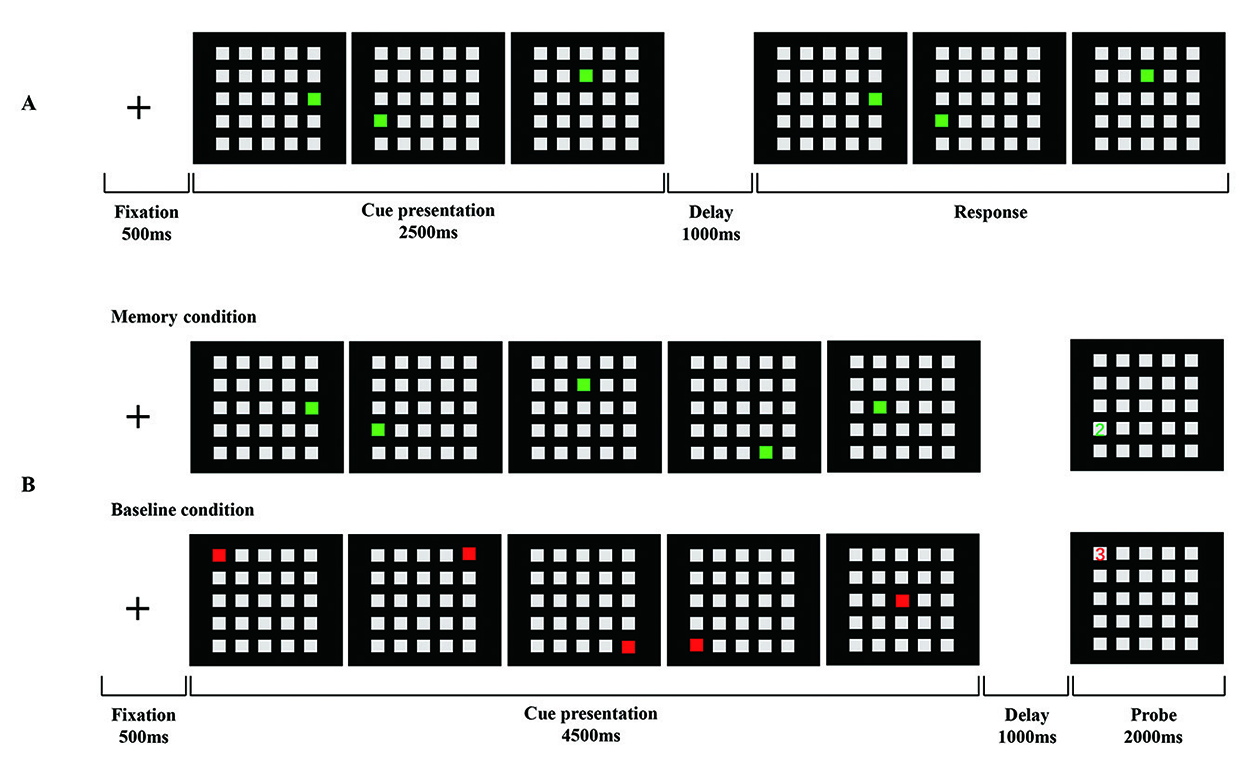

Supplement: Supplementary file 2 [file BRB3-10-e01523-s002.tif]
