# Supplementary material for: Evidence for the contribution of COMT gene Val158/108Met polymorphism (rs4680) to working memory training‐related prefrontal plasticity
Source: Brain Behav. 2020 Jan 9;10(2):e01523. doi: 10.1002/brb3.1523 (PMC7010579; doi:10.1002/brb3.1523)
Supplement: Supplementary file 3 [file BRB3-10-e01523-s003.doc]

**Supplementary Material**

**Supplement Table**

Table S1. Demographic variables across COMT Gene Val158/108Met Polymorphism.

|  | Mean ± SD | | F or χ^2^ | *P* |
| --- | --- | --- | --- | --- |
|  | Val/ Val | Met carriers |  |  |
| Number of subjects | 46 | 50 |  |  |
| Gender (male/female) | 9/37 | 11/39 | 0.78 | 0.806 |
| Age (years) | 21.838±2.27 | 22.04±2.47 | 0.13 | 0.721 |
| Education (years) | 15.37±1.96 | 15.76±1.97 | 0.95 | 0.333 |
| IQ^a^ | 130.462±5.73 | 129.32±6.11 | 0.88 | 0.351 |

^a^ Full scale IQ, as measured by Wechsler Adult Intelligence Scale.

**Supplement Figure Legends**

Figure S1. CONSORT diagram of the two training groups.

Figure S2. The training version (Panel A) and the fMRI version (Panel B) of the visual-spatial span task. During training, subjects were required to remember both the location and the order of all stimuli and tap the squares in the empty grid to indicate the locations of them in the order they were presented. The number of stimuli were kept at 3 for the control group. For the adaptive training group, the number of stimuli started with 3 but would be automatically increased by 1 if the subject showed 5 continuous correct responses on the current difficulty level. For the fMRI version, subjects were required to judge if the Arabic number indicated the correct order of the cue stimuli that was presented. Stimuli were green-colored in the memory condition but were red-colored in the baseline condition.
